# Rapid Remission in Peripheral T-Cell Lymphoma of the Nasal Type by the Bortezomib plus CHOP Therapy

**DOI:** 10.1155/2010/403237

**Published:** 2010-12-26

**Authors:** Li-Jun Xue, Xiao-Bei Mao, Xiao-Bei Liu, Quan-Sheng Su, Hong-Ju Yu, Ji-Hong Yang

**Affiliations:** Department of Oncology and Hematology, Jinling Hospital, Nanjing University School of Medicine, 305 East Zhong-shan Road, Nanjing, Jiangsu Province 210002, China

## Abstract

Peripheral T-cell lymphoma (PTCL) is rare and difficult to treat for its high relapse rate. The authors report a case of PTCL of the skin, regarding which clinical and pathological features, treatment, and prognosis were discussed. A 66-year-old woman was admitted with complaints of enlarging erythematous noduloplaques on the right anterior tibial skin for one year and similar lesions on the left for 6 months. Surgical resection of right leg lesion and biopsy of enlarged inguinal lymph nodes histologically indicated a PTCL of the nasal type. The patient was treated by CHOP plus bortezomib, reached complete remission just after two courses of chemotherapy and then received another two as consolidation. The patient remained in remission for 11 months until local relapse. As for cutaneous lesions, detailed lymph node examination and prompt tissue biopsy are judicious choices prior to any medical management. The chemotherapy consisting of bortezomib and CHOP is safe and efficient in PTCL of the skin.

## 1. Introduction

Peripheral T-cell lymphoma (PTCL), accounting for less than 15% of non-Hodgkin's lymphoma worldwide, derives from natural killer or mature T cells (NK/TCL) and often involves the skin primarily or secondarily [1]. PTCL has a relatively higher morbidity rate in Asia and Central/South America, especially in populations infected with human T-cell lymphoma/leukemia virus-1 or Epstein-Barr virus (EBV) [2]. Diagnosis in PTCL has improved with the development of molecular, immunologic, and genetic techniques. However, there is still no consensus about standardized therapy in PTCL, although CHOP (cyclophosphamide, pirarubicin, vincristine, and prednisone) or CHOP-like chemotherapy have been thought as the major regimen [3]. Progress in treatment of PTCL is much slow mainly due to disease rarity, biological heterogeneity, geographic variation, and limited recognition of the disease. To date, the prognosis of most PTCL types is extremely poor with a 5-year survival of 15–30% in the majority of series [1]. In recent years, bortezomib as a novel proteasome inhibitor, has shown good tolerance and therapeutic effect in PTCL besides defined indications for multiple myeloma and mantle cell lymphoma, which is bringing new light to PTCL patients [4, 5].

Here, we present a case of peripheral NK/TCL of the skin with positive EBV infection, in which bortezomib plus CHOP chemotherapy were used and led to rapid improvement and then complete remission (CR) for 11 months until local relapse. We also discussed the diagnosis, prognosis, and bortezomib- or plus CHOP-based treatment of PTCL according to this case and previously published data.

## 2. Case Report

In October 2007, a 66-year-old woman firstly visited a dermatology clinic with chief complaints of erythematous noduloplaques with mild tenderness and pitting edema on the right anterior tibial skin. She was diagnosed with dermatitis and continuously improved under infrared irradiation until April 2008, when similar dermatic lesions occurred on the left anterior tibial area. In September 2008, the reddish-violet noduloplaques on both legs increased to a size of ahen's egg, and the lesion on right leg even ulcerated and bled. The woman was then admitted to our hospital without a history of fever or weight loss. Physical examination showed multiple painless swollen lymph nodes of moderate hardness and limited motion in bilateral inguinal areas, which were assessed by color Doppler ultrasound with the largest one being 19 × 6 micrometer in size. A comprehensive metabolic profile displayed an elevated serum level of lactate dehydrogenase (LDH) of 256 U/L (normal: 60 to 240 U/l). Computerized tomography scan of the chest and abdomen was normal except for hepatic cysts. 

**Figure 1:**
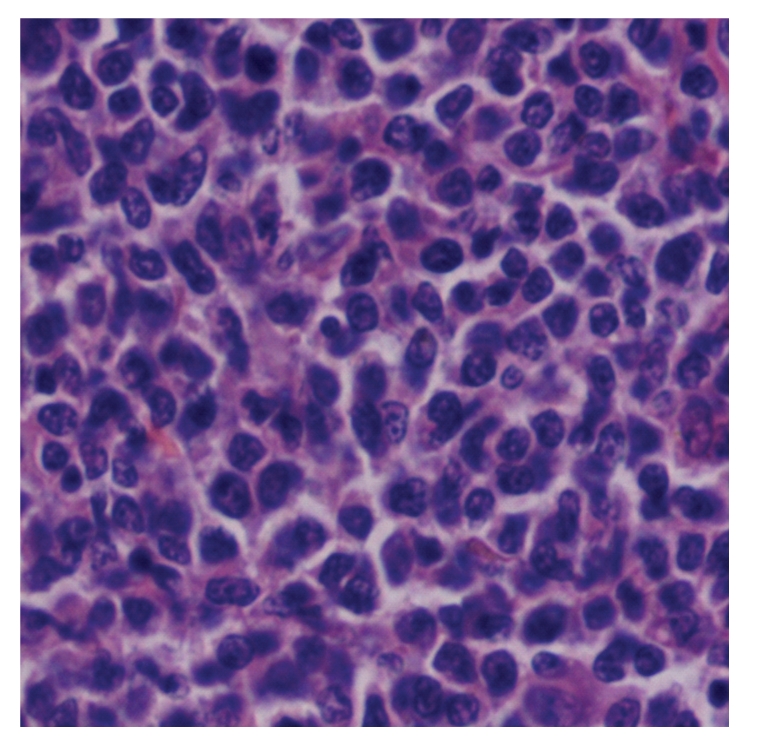
Sections of invaded skin showing prominent infiltration by peripheral natural killer/T-cell lymphoma cells (hematoxylin and eosin, ×400).

**Figure 2:**
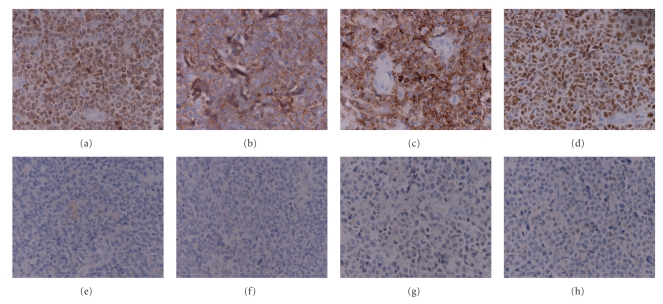
Sections of invaded skin and lymph nodes displaying strongly positive CD3 (cytoplasmic) (a), CD56 (b), CD43 (c), and Ki67 (d), but negative CD20 (e), CD30 (f), Bcl-2 (g), and perforin (h) by immunohistochemistry (×400).

The surgical resection and dermatoplasty of right leg lesion and biopsy of homolateral inguinal lymph nodes were carried out by plastic surgeons. Both the sections of invaded skin and lymph nodes histologically indicated peripheral NK/TCL of the nasal type with strongly positive CD56, CD43, CD3 and Ki67, but negative CD20, CD30, Bcl-2, and perforin by immunohistochemistry (Figures 1 and 2). EBV-encoded RNA (EBER) was positive in skin specimen by in situ hybridization. Serum anti-EBV capsid antigen (CA) IgM and antiearly antigen IgG were negative, but antinuclear antigen-1 IgG, anti-CA IgG, and anti-CA IgA were significantly positive. 

**Figure 3:**
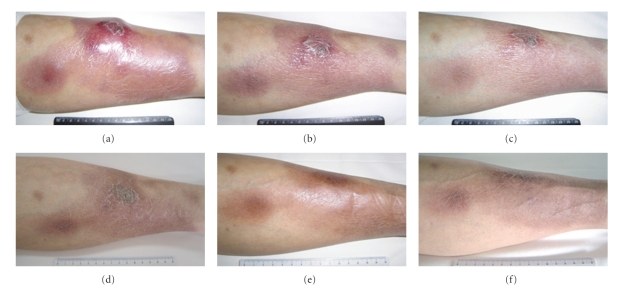
Rapid remission in left leg lesions by bortezomib plus CHOP chemotherapy. (a) Day 0 in the first course. (b) Day 5 in the first course. (c) Day 8 in the first course. (d) Day 5 after the first course. (e) Day 7 after the second course. (f) Day 7 after the fourth course.

The patient underwent a combination chemotherapy consisting of classical CHOP-21 plus bortezomib (1.3 mg/m^2^), which were administered by bolus injection on days 1, 4, 8, and 11, every 21 days. Simultaneously, acyclovir was intravenously used to protect against mucocutaneous infection with herpes simplex virus. The disease was quickly and significantly improved, which mainly displayed in diminishing noduloplaques, shrinking erythemas and swollen lymph nodes, decreasing edema, elimination of tenderness, and normalization of local skin temperature. She reached CR with mild brown pigmentation just after two courses of chemotherapy and received another two courses as consolidation (Figure 3). The therapy was well tolerated with only grade-two toxicities of neutropenia and thrombocytopenia. The patient remained in remission for 11 months but relapsed locally and became lost to followup at last.

## 3. Discussion

As for most PTCL patients with skin involvement but no B symptoms, cutaneous lesions are always significant enough to be initially identified and treated as common dermatitis at a dermatology clinic such as in this case. Detailed physical examination, especially lymph node examination, and prompt tissue biopsy are judicious choices prior to any medical management in order to avoid misdiagnosis and delay of correct treatment. Histologically, PTCL contains multiple subtypes including PTCL unspecified (PTCL-U), cutaneous TCL (CTCL), angioimmunoblastic TCL (AILT), anaplastic large-cell lymphoma (ALCL) noncutaneous, and hepatosplenic TCL (HSTCL) [2]. Adequate immunophenotyping is essential to exclude B-cell lymphomas and establish the specific type of PTCL by immunohistochemistry or flow cytometry. In our case, the immunostaining results of strongly positive CD3, CD43, CD56, and Ki67, but negative CD20, CD30, and Bcl-2, definitely supported the diagnosis of peripheral NK/TCL of the nasal type, although the negativity for perforin is indeed unusual [6].

Up to now, there is no standard efficient therapy for PTCL [1]. CHOP-like treatment, as the most commonly used first-line regimen for PTCL patients, is largely ineffective with low CR and high relapse rate except in anaplastic-lymphoma-kinase- (ALK)-positive ALCL [3, 7]. Some emerging evidence demonstrated that CHOP might not be appropriate as a chemotherapy backbone for PTCL patients since anthracyclines perhaps cannot influence the outcome, at least of PTCL-U [1, 8–10]. Previous data also suggested that expression of P-glycoprotein in PTCL possibly results in multidrug resistance to conventional systemic therapy, at least partly [11]. Other regimens that are more intensive than CHOP did not show any significant improvement in the overall survival of PTCL patients yet, with the exception of ALCL [12].

Bortezomib exerts an antitumor activity mainly through affecting the ubiquitin-proteasome pathway subsequently leading to inhibition of NF-*κ*B and stabilization of proapoptotic proteins such as P53, Bcl-2, Bim, Bik, and Noxa [13]. It has been reported that NF-*κ*B plays a critical role in the pathogenesis of CTCL, and that bortezomib can significantly cause NF-*κ*B down-regulation and CTCL cell apoptosis [14]. Therefore, bortezomib may play a special therapeutic role and, at the same time, be safe from the emergence of acute toxicity in some types of PTCL due to its basic function mechanisms. As reported, an advanced HSTCL patient of gamma/delta type remained almost in CR for 27 months after 4 cycles of bortezomib plus the modified high-dose CHOP prior to autologous peripheral blood stem cell transplantation [15]. A phase Ⅱ clinical trial has confirmed that bortezomib indeed is safe and has significant single-agent activity in inducing remission (67% overall response rate, ORR), even CR (17%), in patients with relapsed or refractory CTCLs including mycosis fungoides and PTCL-U with isolated skin involvement [4]. The mentioned study also indicated that bortezomib leads to higher or at least comparable ORR compared with published data on gemcitabine, pegylated liposomal doxorubicin, 2-chlorodeoxyadenosine, or pentostatin. Another recent phase I study demonstrated that PTCL patients consisting of PTCL-U, NK/TCL, ALCL, and angioimmunoblastic lymphoproliferative disease gained profits from the bortezomib plus CHOP therapy, with 61.5% ORR and unconfirmed CR rates [5]. The combined regimen just caused grade-4 neutropenia associated with febrile episode in one patient and grade-one peripheral sensory neuropathy in three patients, which indicated a good tolerance. 

It has been reported that PTCL-U presenting in the skin has an unfavorable prognosis [16]. The outcome of PTCL appears to be associated with the International Prognostic Index (IPI), Prognostic Index for PTCL-U (PIT), and the International peripheral T-cell lymphoma Project score (IPTCLP), which are used for risk classification and response and survival prediction [3, 12, 17, 18]. In addition, EBV is positive in about 40% of PTCL, and some case series have reported that EBER-positive tumors have a worse outcome [3, 19]. Our patient belongs to the subgroup of high risk with bad prognosis because of poor prognostic indexes due to multiple risk factors: >60 years of age, ECOG performance status >2, late stage, elevated serum LDH, number of extranodal sites of involvement >1, high Ki67 expression, and positive EBV infection [20, 21]. Anyway, the adopted regimen showed enough safety and quickly led to satisfied therapeutic effects in this case. Therefore, the bortezomib plus CHOP chemotherapy is safe and efficient in peripheral NK/TCL according to our case and the literature review, although further large-sized clinical trials with long-period followup are still needed.
